# Phase 1 trial of entinostat as monotherapy and combined with exemestane in Japanese patients with hormone receptor-positive advanced breast cancer

**DOI:** 10.1186/s12885-021-08973-4

**Published:** 2021-11-24

**Authors:** Norikazu Masuda, Kenji Tamura, Hiroyuki Yasojima, Akihiko Shimomura, Masataka Sawaki, Min-Jung Lee, Akira Yuno, Jane Trepel, Ryoko Kimura, Yozo Nishimura, Shigehira Saji, Hiroji Iwata

**Affiliations:** 1 Department of Surgery, Breast Oncology, National Hospital Organization Osaka National Hospital, Osaka, Japan; 2 Present address: Department of Breast and Endocrine Surgery, Nagoya University Graduate School of Medicine, 65 Tsurumai-cho, Showa-ku, Nagoya, 466-8550 Japan; 3 Department of Breast and Medical Oncology, National Cancer Center Hospital, Tokyo, Japan; 4 Present address: Department of Medical Oncology, Shimane University Hospital, Izumo, Shimane Japan; 5 Present address: Department of Breast and Medical Oncology, National Center for Global Health and Medicine, Tokyo, Japan; 6 Department of Breast Oncology, Aichi Cancer Center Hospital, Nagoya, Aichi Japan; 7 Developmental Therapeutics Branch, Center for Cancer Research, National Cancer Institute, National Institutes of Health, Bethesda, MD USA; 8 Present address: Department of Oral and Maxillofacial Surgery, Kumamoto University Hospital, Kumamoto, Japan; 9 R&D Division, Kyowa Kirin Co., Ltd., Tokyo, Japan; 10 Department of Medical Oncology, Fukushima Medical University, Fukushima, Japan

**Keywords:** Acetylation, Aromatase inhibitors, Drug resistance, Epigenomics, Histone deacetylases

## Abstract

**Background:**

Entinostat is an oral inhibitor of class I histone deacetylases intended for endocrine therapy-resistant patients with hormone receptor-positive (HR+) advanced or metastatic breast cancer (BC). We examined the safety, efficacy, and pharmacokinetics of entinostat monotherapy and combined entinostat/exemestane in Japanese patients.

**Methods:**

This phase 1 study (3 + 3 dose-escalation design) enrolled postmenopausal women with advanced/metastatic HR+ BC previously treated with nonsteroidal aromatase inhibitors. Dose-limiting toxicities (DLTs) of entinostat monotherapy (3 mg/qw, 5 mg/qw, or 10 mg/q2w) and entinostat+exemestane (5 mg/qw + 25 mg/qd) were assessed. Pharmacokinetics, lysine acetylation (Ac-K), and T-cell activation markers were measured at multiple time points.

**Results:**

Twelve patients were enrolled. No DLTs or grade 3–5 adverse events (AEs) occurred. Drug-related AEs (≥ 2 patients) during DLT observation were hypophosphatemia, nausea, and platelet count decreased. Six patients (50%) achieved stable disease (SD) for ≥ 6 months, including one treated for > 19 months. Median progression-free survival was 13.9 months (95% CI 1.9–not calculable); median overall survival was not reached. Area under the plasma concentration-time curve and Ac-K in peripheral blood CD19+ B cells increased dose-proportionally. The changing patterns of entinostat concentrations and Ac-K levels were well correlated. T-cell activation markers increased over time; CD69 increased more in patients with SD ≥ 6 months vs. SD < 6 months.

**Conclusions:**

Entinostat monotherapy and combined entinostat/exemestane were well tolerated in Japanese patients, with no additional safety concerns compared with previous reports. The correlation between pharmacokinetics and Ac-K in peripheral blood CD19+ B cells, and also T-cell activation markers, merits further investigation.

**Trial registration:**

JAPIC Clinical Trial Information, JapicCTI-153066. Registered 12 November 2015. ClinicalTrials.gov, NCT02623751. Registered 8 December 2015.

**Supplementary Information:**

The online version contains supplementary material available at 10.1186/s12885-021-08973-4.

## Background

The goal of treatment for patients with advanced or metastatic breast cancer (BC) is to control the disease, prolong survival, and improve quality of life. In hormone receptor-positive (HR+) and human epidermal growth factor receptor 2-negative (HER2−) BC, sequential hormonal therapies targeting estrogen signaling are the standard treatment. Currently, monotherapy with a nonsteroidal aromatase inhibitor (NSAI), a selective estrogen receptor degrader like fulvestrant, or combination therapy with an NSAI and a cyclin-dependent kinase (CDK) inhibitor [1–6] are commonly used as first-line therapy. Second- or third-line therapies include monotherapy with a steroidal aromatase inhibitor, fulvestrant, tamoxifen, or combination therapies with a hormonal therapy and a molecular targeted therapy, such as everolimus, a mammalian target of rapamycin (mTOR) inhibitor [7], or a CDK4/6 inhibitor [1, 5, 6]. Each combination therapy has demonstrated a significant prolongation of progression-free survival (PFS) compared with hormonal monotherapy [1, 5–7]. In addition, CDK4/6 inhibitors showed a statistically significant prolongation of overall survival (OS) as a secondary endpoint when used in combination with fulvestrant [8–11]. However, there is no standard care for cases of disease progression after endocrine-based therapy with a CDK4/6 inhibitor. Therefore, there is a need for new agents that can be combined with endocrine therapy.

Histone deacetylases (HDACs) are chromatin-modifying enzymes that have both direct and indirect effects on gene transcription, primarily via deacetylation of lysine residues of histones. Administration of HDAC inhibitors (HDACi) can cause global gene expression changes, with 7–10% of gene expression up- or down-regulated [12–17]. HDAC isoforms are grouped into four classes according to molecular structure and function [18]. Entinostat (ENT) is an orally available benzamide-type class I specific HDACi. In HR+ BC, it is reported that reduced expression of estrogen receptor (ER) and activation of alternative signaling pathways contribute to hormonal therapy resistance in some cases [19]. ENT is considered to improve sensitivity to hormonal therapies by promoting re-expression of ER and inhibiting alternative signaling pathways [19].

In a double-blind, placebo-controlled, phase 2 study (ENCORE 301, *N* = 130) of ENT in combination with exemestane (EXE) in patients with HR+/HER2− advanced or metastatic BC, the ENT + EXE group showed improved PFS (median 4.3 vs. 2.3 months; hazard ratio [HR] 0.73; 95% CI 0.50–1.07; one-sided *P* = 0.055) and OS (median 28.13 vs. 19.84 months; HR 0.59; 95% CI 0.36–0.97; one-sided *P* = 0.018) compared with placebo + EXE [20]. Although OS was an exploratory endpoint, prolongation of OS drove the US Food and Drug Administration to designate ENT as a breakthrough therapy. Currently, a global, double-blind, phase 3 study with PFS and OS as co-primary endpoints (E2112, *N* = 600, NCT02115282) and a Chinese phase 3 study with PFS as primary endpoint (*N* = 327, NCT03538171) are being conducted in patients with HR+/HER2− advanced or metastatic BC.

ENT has already been administered to more than 1000 healthy subjects and patients worldwide, but no phase 1 study has yet been conducted in Asian patients. Therefore, we performed this study to obtain data on the safety, pharmacokinetics (PK), protein lysine acetylation (Ac-K) levels of peripheral blood mononuclear cells (PBMCs), and T-cell activation markers in Japanese patients with HR+/HER2− BC treated with ENT as monotherapy and in combination with EXE. In addition, to our knowledge, this is the first trial not only to explore the dose-dependent effect of ENT using non-weight-based dosing, but also to compare the changing patterns between ENT concentrations and Ac-K levels and to assess markers of T-cell activation.

## Methods

### Study design

This study was based on a 3 + 3 dose-escalation design (Additional file 1). The dose-limiting toxicities (DLTs) of ENT monotherapy (3 mg or 5 mg once weekly or 10 mg as a single dose) in Cohorts 1–3 and ENT 5 mg weekly in combination with EXE 25 mg/d in Cohort 4 were assessed for 7 days (cycle 0) and 28 days (cycle 1), respectively. Doses of ENT were set as 3, 5 (the expected global clinical dose), and 10 mg in Cohorts 1–3 to determine the relationship between dose and PK parameters in Japanese patients. Patients continued ENT (3 mg or 5 mg) in combination with EXE after the DLT observation period in Cohorts 1, 2, and 4. In Cohort 3, weekly administration of ENT 5 mg started 2 weeks after the single administration of ENT 10 mg, whereas EXE was initiated 1 week after the first ENT administration. The duration of cycle 0 was 7 days and that of each cycle after cycle 1 was 28 days in principle. Treatment continued until progressive disease (PD) or discontinuation for other reasons (Additional file 1).

The study was approved by the institutional review board at each participating site and conducted in accordance with the principles of Good Clinical Practice and the Declaration of Helsinki.

### Eligibility

Postmenopausal women aged 20–74 years at the time of providing written informed consent, with unresectable advanced or metastatic HR+/HER2− BC previously treated with NSAIs and with Eastern Cooperative Oncology Group performance status (ECOG PS) 0–1 who were scheduled to receive EXE, were eligible. Patients should not have previously received more than three regimens of chemotherapy for advanced or metastatic BC. Previous treatment with fulvestrant or a CDK4/6 inhibitor was allowed, although CDK4/6 inhibitors were not approved in Japan when the trial started. Patients with measurable lesions (including patients with only bone lesions) according to the Response Evaluation Criteria in Solid Tumours (RECIST) version 1.1 evaluation criteria were eligible for inclusion. Postmenopause is defined as no menses for ≥ 12 consecutive months and age ≥ 55 years, amenorrheic for ≥ 1 year and age < 55 years together with a blood estradiol level < 20 pg/mL, or surgical menopause with bilateral oophorectomy. Women in whom ovarian function has been suppressed by irradiation of the ovaries or treatment with luteinizing hormone-releasing hormone agonists (goserelin acetate or leuprorelin acetate) were ineligible.

### Endpoint and assessment

The primary endpoint was safety. The severity of adverse events (AEs) was evaluated using National Cancer Institute Common Terminology Criteria for Adverse Events version 4.03 [21]. Electrocardiogram data were collected throughout the study period, and change in QTc interval from baseline was evaluated after the first administration of ENT.

The secondary endpoints were PK and efficacy. Plasma ENT concentrations were measured and PK parameters were evaluated. Antitumor effects and PFS were evaluated in the efficacy assessment. Tumor sizes were measured every 8 weeks (two cycles) for 12 months and then every 12 weeks (three cycles) thereafter by computed tomography or magnetic resonance imaging. Antitumor effects were evaluated according to RECIST version 1.1 [22].

### Definition of DLTs

Among the AEs that occurred during the DLT observation period, those meeting the following criteria at the end of this period were defined as DLTs: grade 4 neutrophil count decreased lasting ≥ 1 week (because grade 4 neutropenia can usually be managed by temporary suspension of ENT, the trial allowed suspension once during the 7-day DLT evaluation period, and any grade 4 neutropenia that lasted ≥ 1 week was considered a DLT); anemia requiring red blood cell transfusion; grade 4 platelet count decreased or platelet count decreased requiring platelet transfusion; neutrophil count < 500/μL and fever ≥ 38.0 °C observed on the same day; and grade ≥ 3 nonhematological toxicities excluding nausea, vomiting, diarrhea, or hypophosphatemia occurring in patients without prophylactic treatment. In addition, the following were also defined as DLTs: AEs resulting in ≥ 2 interruptions in one cycle or discontinuation of ENT in the next cycle; increased hazards to patients that were considered serious; or ENT dose reduction that was required in the opinion of the investigator.

### Criteria for dose interruption/reduction

When grade ≥ 3 AEs were observed for which the causal relationship with ENT and/or EXE could not be ruled out, treatment with ENT and/or EXE was interrupted for a maximum of 2 weeks until these events returned to grade 1 or baseline. However, patients with nausea, vomiting, diarrhea, and hypophosphatemia were allowed to continue treatment without interruption by receiving the appropriate care. Regarding hematological toxicity, treatment with ENT and EXE was resumed after the AE recovered to grade 2 as long as the investigator was satisfied that patient safety could be guaranteed.

### Statistical analysis

The safety analysis set (to evaluate safety) and the full analysis set (to evaluate efficacy) included all of the enrolled patients, except those who did not receive the study drug. AEs were summarized by System Organ Class and Preferred Term according to the Medical Dictionary for Regulatory Activities, version 20.1. The Kaplan-Meier method was used to estimate the median PFS (mPFS). PFS is defined as the duration of time from day 1 to the date of death or of PD, whichever came first. Patients who received subsequent therapy before confirmation of PD or in whom neither PD nor death was confirmed were censored at the last time point of non-PD confirmation. Clinical benefit rate (CBR) was defined as the percentage of patients achieving complete response (CR) or partial response (PR), or who maintained stable disease (SD) for more than 6 months.

### PK

Plasma ENT concentration was measured intensively on cycle 0, day 1 and cycle 1, day 1 in Cohorts 1 and 2 and cycle 1, day 1 in Cohort 4. Thereafter, it was measured before ENT administration on cycle 0, days 2, 4, and 6 and cycle 1, day 1 in Cohorts 1 and 2; in Cohorts 1, 2, and 4 it was measured on cycle 1, days 2, 4, 6, 8, 15, and 22 and cycle 2, day 1. In Cohort 3, plasma ENT concentration was measured intensively on cycle 0, day 1. Thereafter, it was measured before ENT administration on cycle 0, days 2, 4, and 6 and cycle 1, days 1, 4, 8, 15, and 22, as well as on cycle 2, day 1. The PK parameters assessed after single and/or combination therapy included time at which maximum plasma concentration (C_max_) was observed (t_max_), and the area under the plasma concentration-time curve from time 0–168 h (AUC_0–168_). Plasma ENT concentrations were measured using a liquid chromatography/tandem mass spectrometry method.

### Ac-K

Ac-K levels in PBMCs were measured as a potential pharmacodynamic biomarker of ENT. In Cohorts 1–3, the samples were collected before ENT administration on cycle 0, days 1 and 2; cycle 1, days 1 and 8; and cycle 2, day 1. In Cohort 4, the samples were collected before ENT administration on cycle 1, days 1, 8, and 15 and cycle 2, day 1. Ac-K levels in CD19+ cells, CD56+ CD16- cells, CD14+ monocytes, CD16+ cells, CD3+ T-cells, and CD45+ cells were measured using the previously reported methods [20, 23], with a modification where antiacetylated lysine monoclonal antibody (BioLegend, San Diego, CA, USA; Clone 15G10, Cat. 623406) was used.

### T-cell activation markers

T-cell activation markers were measured in Cohorts 1–3 (cycle 0, days 1 and 2; cycle 1, days 1 and 8; and cycle 2, day 1) to investigate the pharmacological action of ENT. Staining of CD3+ cells was performed using antibodies (all from BioLegend) against CD4 (Clone RPA-T4, Cat. 300512), CD8 (Clone SK-1, Cat. 344718), CD69 (Clone FN50, Cat. 310910), inducible T-cell costimulator (ICOS; Clone C398.4A, Cat. 313514), and human leukocyte antigen-DR (HLA-DR; Clone L243, Cat. 307630).

## Results

The study population consisted of 12 patients. Three patients were assigned to each of four cohorts (Cohorts 1–4) between November 2015 and September 2016. All 12 patients were included in the safety and efficacy analyses. At the cut-off date of September 2017, four patients continued to receive study treatment. The median age of patients was 61.5 years; seven patients (58.3%) had visceral metastases at baseline and five patients (41.7%) received ≥ 3 regimens of hormonal therapies for advanced or metastatic BC (Table 1). Three patients had received prior NSAIs in the adjuvant setting, and nine patients had received prior NSAIs in the advanced or metastatic setting. Six patients had previously received fulvestrant.

**Table 1:** Demographic and other baseline characteristics

Characteristic	Total	Cohort 1 ENT 3 mg mono → EXE combo	Cohort 2 ENT 5 mg mono → EXE combo	Cohort 3 ENT 10 mg mono → EXE combo	Cohort 4 ENT 5 mg + EXE combo
N	12	3	3	3	3
Age, median (range), years	61.5 (48–72)	56.0 (48–62)	61.0 (59–62)	64.0 (55–65)	68.0 (55–72)
Baseline BMI, mean (SD), kg/m^2^	22.82 (3.25)	24.00 (4.30)	22.53 (4.73)	22.33 (3.08)	22.40 (2.19)
Baseline ECOG PS					
0	11 (91.7)	3 (100.0)	2 (66.7)	3 (100.0)	3 (100.0)
1	1 (8.3)	0	1 (33.3)	0	0
Histopathological description					
Invasive ductal carcinoma	10 (83.3)	3 (100.0)	3 (100.0)	3 (100.0)	1 (33.3)
Other	2 (16.7)	0	0	0	2 (66.7)
ER+/PgR+	10 (83.3)	2 (66.7)	3 (100.0)	3 (100.0)	2 (66.7)
ER+/PgR−	1 (8.3)	0	0	0	1 (33.3)
ER−/PgR+	1 (8.3)	1 (33.3)	0	0	0
Current metastatic site	12 (100.0)	3 (100.0)	3 (100.0)	3 (100.0)	3 (100.0)
Visceral disease (lung, liver metastasis)	7 (58.3)	2 (66.7)	2 (66.7)	2 (66.7)	1 (33.3)
Prior surgery	11 (91.7)	3 (100.0)	3 (100.0)	2 (66.7)	3 (100.0)
Prior systemic therapy^a^	11 (91.7)	2 (66.7)	3 (100.0)	3 (100.0)	3 (100.0)
Prior chemotherapy^a^					
0	8 (66.7)	2 (66.7)	3 (100.0)	2 (66.7)	1 (33.3)
1	2 (16.7)	1 (33.3)	0	1 (33.3)	0
2	2 (16.7)	0	0	0	2 (66.7)
Prior endocrine therapy^a^					
0	1 (8.3)	1 (33.3)	0	0	0
1	3 (25.0)	1 (33.3)	1 (33.3)	1 (33.3)	0
2	3 (25.0)	0	1 (33.3)	1 (33.3)	1 (33.3)
≥ 3	5 (41.7)	1 (33.3)	1 (33.3)	1 (33.3)	2 (66.7)

All values are n (%), unless otherwise stated

*BMI* Body mass index, *ECOG PS* Eastern Cooperative Oncology Group performance status, *ENT* Entinostat, *ER+/−* estrogen receptor-positive/negative, *EXE* Exemestane, *PgR+/−* Progesterone receptor-positive/negative, *S.D.* Standard deviation

^a^For advanced or metastatic breast cancer

### Safety

In the DLT observation period, no DLTs or grade 3–5 AEs occurred. The drug-related AEs observed in ≥ 2 patients during the DLT observation period were grade 1 or 2 hypophosphatemia (one patient in Cohorts 2, 3, and 4), grade 1 nausea (one patient in Cohort 3 and two patients in Cohort 4), and grade 1 or 2 platelet count decreased (two patients in Cohort 4).

During the DLT observation period, ENT dose reduction was not observed in any of the cohorts. When ENT was administered in combination with EXE after the completion of the DLT observation period, one patient in Cohort 2 and two patients in Cohort 4 required a dose reduction from 5 mg to 3 mg of ENT owing to platelet count decreased and hypophosphatemia.

All drug-related AEs observed in ≥ 2 patients from the start of the study to the cut-off date are shown in Table 2. The most frequently reported AE as of the cut-off date was neutrophil count decreased in five patients with grade 2 and in three patients with grade 3 AEs. The grade 3 AEs observed were hypophosphatemia for four patients (Cohorts 2–4), neutrophil count decreased for three patients (Cohorts 1, 2, and 4), platelet count decreased for two patients (Cohorts 2 and 4), and anemia for one patient (Cohort 4).

**Table 2:** Drug-related AEs developed in ≥ 2 patients as of data cut-off date

PT [SOC]^a^	Total *N* = 12				Cohort 1 *N* = 3				Cohort 2 *N* = 3				Cohort 3 *N* = 3				Cohort 4 *N* = 3			
	G1	G2	G3	G4	G1	G2	G3	G4	G1	G2	G3	G4	G1	G2	G3	G4	G1	G2	G3	G4
Subjects with any drug-related AE		4	8			2	1				3			1	2			1	2	
[Blood and lymphatic system disorders]																				
Anemia	1	2	1			1			1					1					1	
[Gastrointestinal disorders]																				
Nausea	6								2				2				2			
Diarrhea	1	1				1			1											
Gastritis	2								1								1			
[General disorders and administration site conditions]																				
Fatigue	3								1				1				1			
[Investigations]																				
Neutrophil count decreased		5	3			2	1			1	1			1				1	1	
Platelet count decreased	4	1	2		2				1		1		1					1	1	
White blood cell count decreased	1	4			1	1				1				1				1		
Weight decreased	1	1				1											1			
[Metabolism and nutrition disorders]																				
Hypoalbuminemia	2	5			1	1				1				2			1	1		
Hypophosphatemia		1	4								2			1	1				1	
Decreased appetite	2								1				1							
[Musculoskeletal and connective tissue disorders]																				
Back pain	2				1								1							
[Nervous system disorders]																				
Headache	2								1				1							
[Respiratory, thoracic and mediastinal disorders]																				
Pneumonitis	2				1												1			

*AE* Adverse event, *G* Grade, *PT* Preferred Term, *SOC* System Organ Class

^a^The listing for AEs displaying the SOC and PT

Serious AEs (SAEs) from the start of the study to the cut-off date included one case each of abdominal pain (grade 2), pulmonary embolism (grade 3), maculopapular rash (grade 3), and femoral neck fracture (grade 3). Although a causal relationship with ENT could be ruled out only for the femoral neck fracture, the remaining SAEs resolved, recovered, or improved with appropriate treatment (abdominal pain: treatment with supplements and antiemetics; pulmonary embolism: treatment with anticoagulants; maculopapular rash: treatment with steroids).

ENT had no effect on the electrocardiograms of the patients tested.

### Efficacy

**Fig. 1:**
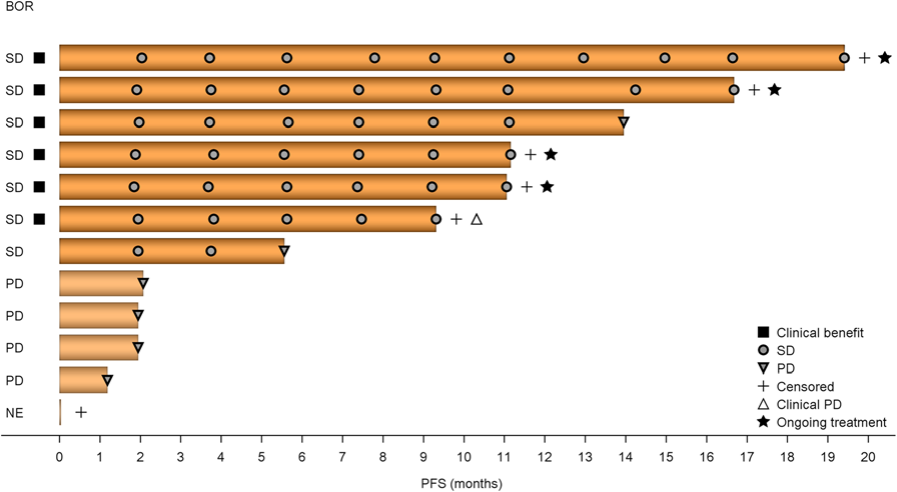
Objective response and PFS. The censored patient did not receive ENT 3 times during Cycle 2 (after the end of the DLT observation period), thereby meeting one of the criteria for discontinuation, namely that patients who did not take ENT three times within one cycle, or who did not take ENT three times in a row regardless of cycle, should be discontinued. The patient then started post-discontinuation treatment before diagnostic imaging was performed to assess possible progression. Therefore, we censored the patient at the time of the previous imaging at study entry. *BOR* best overall response, *DLT* dose-limiting toxicity, *ENT* entinostat, *NE* not evaluable, *PD* progressive disease, *PFS* progression-free survival, *SD* stable disease

As of the cut-off date, six patients discontinued the study drug owing to PD and six patients were censored (one patient with clinical PD, one patient not evaluated, and four patients continuing to receive study treatment, including one patient treated for more than 19 months). No patient achieved CR or PR as best response; therefore, the objective response rate was 0%. SD for > 6 months was achieved in six patients; therefore, CBR was 50.0%. The mPFS was 13.9 months (95% CI 1.9–not calculable) (Fig. 1). Median OS was not reached.

### PK

**Fig. 2:**
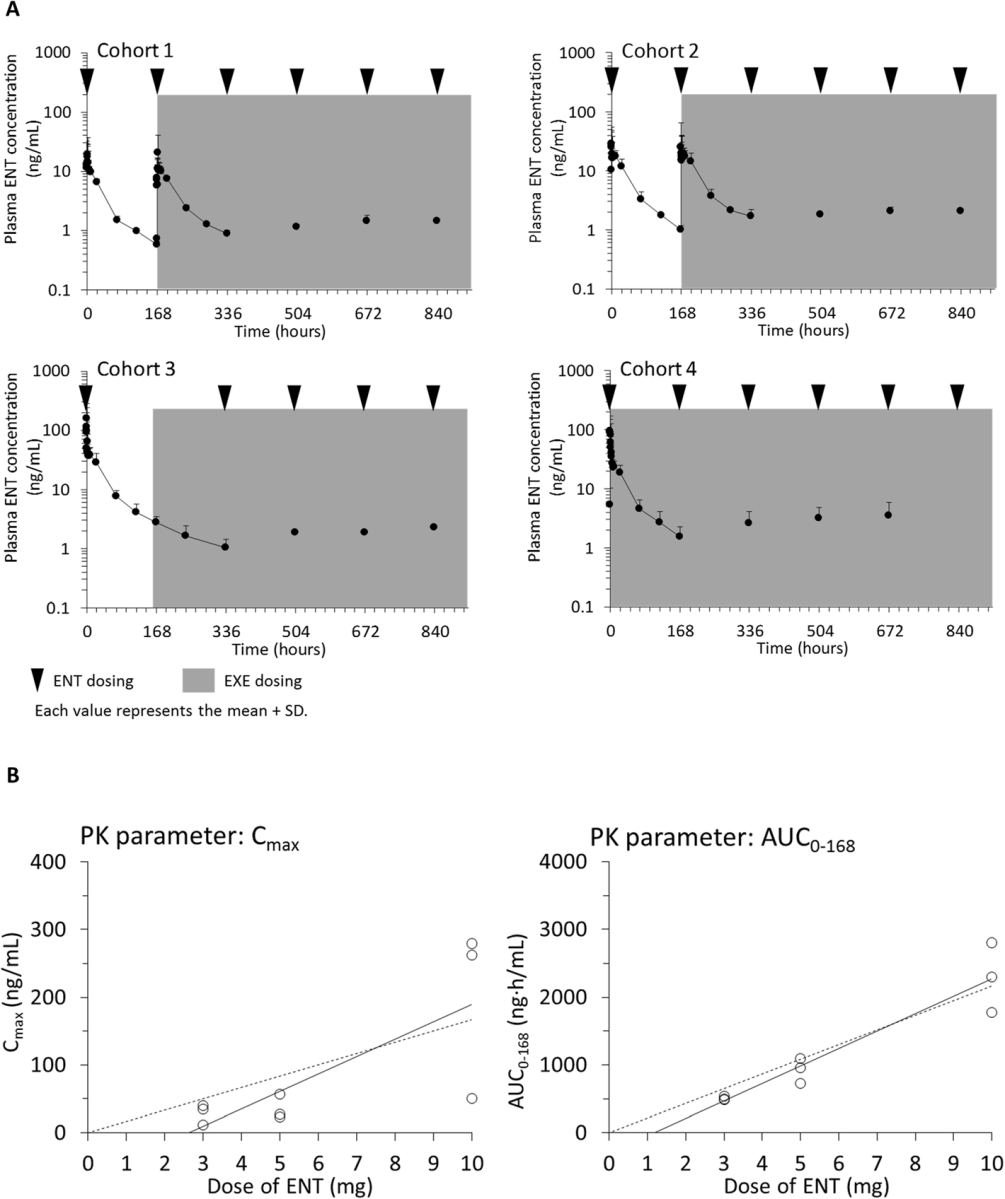
Plasma ENT concentration-time profiles and PK parameters of ENT. **a** Plasma ENT concentration-time profiles in each cohort. Each point represents the mean + S.D. ENT was administered at points of reverse triangle in combination with EXE 25 mg once daily during the shaded time period. **b** Dose proportionality of PK parameters of ENT. Each point represents individual subject value after the first administration of ENT. Linear regression lines are superimposed with fixing (broken line) and without fixing (solid line) the y-intercept to origin. *AUC*_*0–168*_ area under the plasma concentration-time curve from time 0–168 h, *C*_*max*_ maximum plasma concentration, *ENT* entinostat, *EXE* exemestane, *PK* pharmacokinetic, *S.D.* standard deviation

Results of the PK evaluation common to all cohorts showed that ENT was rapidly absorbed following oral administration (t_max_ 0.48–1.42 h) and reached a steady state on cycle 1, day 15 (Fig. 2a). The PK profiles of ENT monotherapy were similar to those of ENT + EXE combination therapy, suggesting that EXE had no influence on the PK of ENT. The C_max_ was increased in accordance with dose escalation between 3 mg and 5 mg, but intersubject variability was high at 10 mg. The AUC_0–168_ following administration of ENT alone increased linearly with dose (Fig. 2b).

### Ac-K

**Fig. 3:**
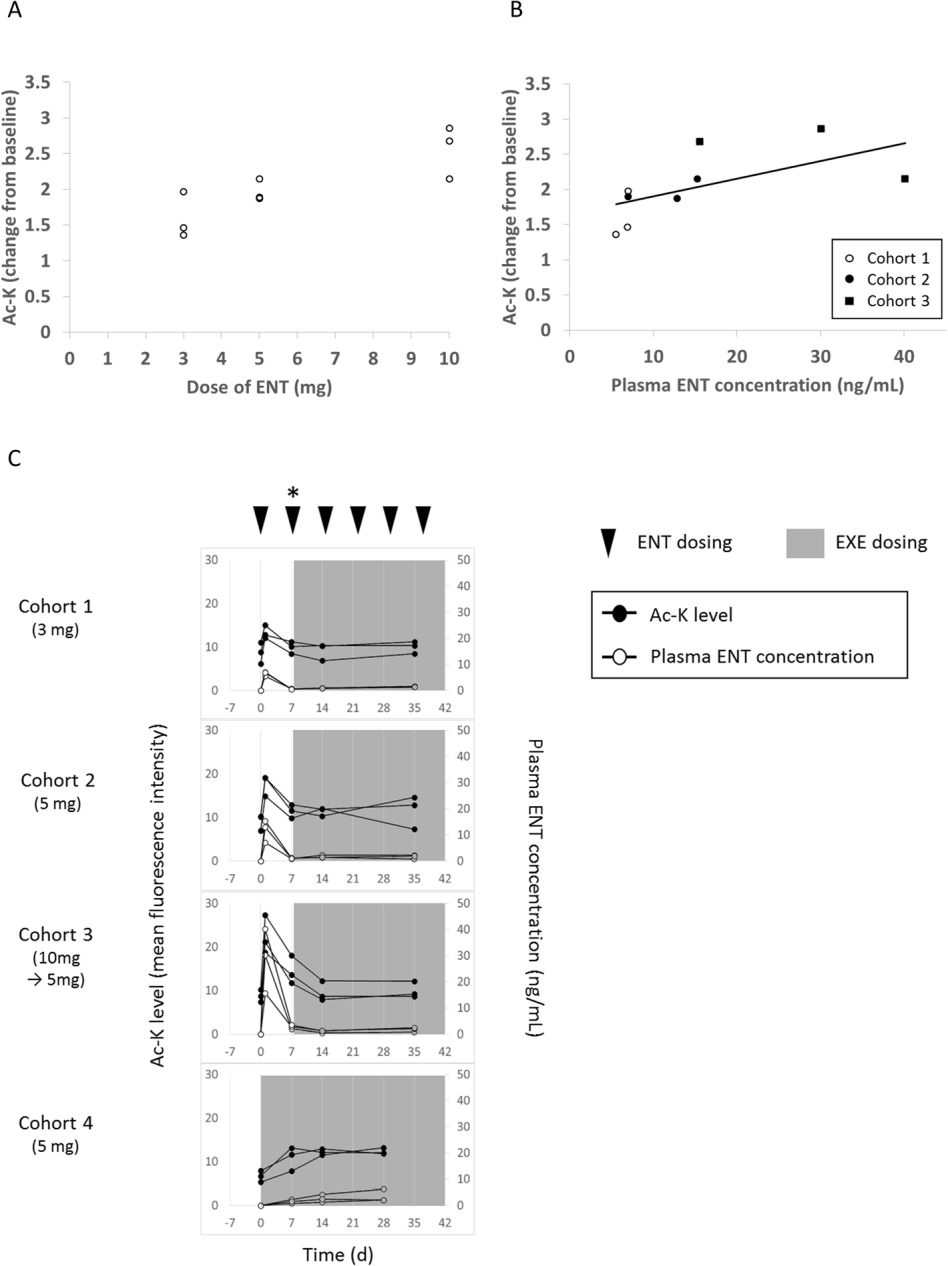
Dose proportionality of Ac-K parameters of ENT and the relationship between PK and Ac-K. Profiles of Ac-K levels in CD19+ B cells and plasma ENT concentration. **a** Dose proportionality of Ac-K levels in CD19+ B cells. Each open circle represents the Ac-K level (change from baseline) in each patient. **b** Relationship between PK and Ac-K levels in CD19+ B cells. Each open circle, filled circle, and filled square represents each Ac-K level (change from baseline) in Cohort 1, Cohort 2, and Cohort 3, respectively. **c** Time course of Ac-K levels and plasma ENT concentration in each cohort. Each filled circle represents Ac-K level (median fluorescence intensity) and each open circle represents plasma ENT concentration (ng/mL). In Cohort 3, ENT was not administered at a dosing point marked with an asterisk (*). Each peripheral blood mononuclear cell sample was collected before ENT/EXE administration. *Ac-K* lysine acetylation, *ENT* entinostat, *EXE* exemestane, *PK* pharmacokinetics

Ac-K levels in CD19+ cells were increased in an ENT dose-dependent manner by about 1.5-fold in Cohort 1, 1.9-fold in Cohort 2, and 2.7-fold in Cohort 3 at 24 h after first administration of study drug compared with the levels prior to administration (Fig. 3a). In addition, Ac-K levels in PBMCs measured 24 h after first administration of ENT were increased in a plasma ENT concentration-dependent manner (Fig. 3b). Ac-K levels in PBMCs were measured multiple times, including 24 h after first administration of ENT when plasma ENT concentrations were relatively high, to monitor dynamic changes in Ac-K (Fig. 3c). Similar trends were observed in the other immune subsets tested (data not shown).

### T-cell activation markers

In Cohorts 1–3, the expression of HLA-DR in CD4+ and CD8+ T-cells increased over time with ENT treatment (Additional file 2). Increased expression of CD69 was also observed in some patients, and the increase tended to be greater in patients with SD for ≥ 6 months compared with patients whose disease progressed in < 6 months (Additional file 3).

## Discussion

### Safety

In a previous phase 1 study of ENT monotherapy for refractory solid tumors and lymphoid malignancies, asthenia (ENT 2 mg/m^2^ twice weekly) and hypophosphatemia and asthenia (ENT 5 mg/m^2^ once weekly) occurred as DLTs [24]. In that study, 4 mg/m^2^ once weekly was determined to be the maximum tolerated dose. Because we did not observe any DLTs with doses that were lower than the dose at which DLT was observed in the previous study, these results are considered consistent without the need to formally determine the maximum tolerated dose in Japanese patients. It should be noted that the equivalent dose and dosage (5 mg weekly) were also used in ENCORE 301 and are currently being used in ongoing clinical trials in the USA and elsewhere, with no safety concerns [25].

In this study, the AEs observed throughout the study period were consistent with previous clinical experience of HDACi, including ENCORE 301 [20, 26–33], and no unique AEs in Japanese patients were observed. AEs that occurred by the data cut-off date and resulted in an ENT dose reduction included platelet count decreased and hypophosphatemia; however, these AEs were manageable with dose interruption for up to 2 weeks followed by dose reduction. Three patients interrupted study treatment owing to grade 3 neutrophil count decreased and two patients because of grade 3 platelet count decreased. However, they interrupted treatment with ENT and then resumed treatment. Grade 3 hypophosphatemia occurred in four patients; however, the AEs were manageable with oral phosphorus supplementation, dose interruption, and dose reduction. In conclusion, ENT in combination with EXE, at the recommended dose assessed in the ENCORE 301 study [20], was generally safe for Japanese patients with BC and was manageable with appropriate measures, such as dose interruption and reduction as well as prophylactic treatment. However, additional studies with larger sample sizes are required to confirm the safety of this dosing regimen in Japanese patients.

### Ac-K

HDACi are considered to exert a therapeutic effect by promoting increased acetylation of lysine residues of intracellular histone and nonhistone proteins, resulting in a global change in gene expression and signal transduction in various ways. The extent of intracellular “hyperacetylation” of histones or nonhistone proteins induced by HDACi in human PBMCs has been examined in clinical trials as a potential pharmacodynamic marker.

In ENCORE 301, total Ac-K levels in PBMCs were measured before and 2 weeks after the first administration of ENT as a trough level (ie, 1 week after the second administration and before the third administration). This analysis demonstrated that PFS tended to be longer in hyperacetylated patients compared with nonhyperacetylated patients, indicating the potential of Ac-K levels as a pharmacodynamic biomarker [20]. This hypothesis is being tested as an integrated biomarker in the ongoing phase 3 study. In this phase 1 study, we measured Ac-K levels in PBMCs at different time points to further investigate their clinical meanings. This study is the first to analyze correlations between total Ac-K levels in PBMCs and ENT levels in blood in patients with advanced or metastatic BC, demonstrating that the changing patterns of Ac-K levels were well correlated with blood ENT concentrations. Ac-K levels were increased 24 h after administration and had decreased by the next administration. Nevertheless, the Ac-K levels of some patients seemed to be up-regulated continuously after administration in comparison to the baseline level.

A clear relationship was not apparent between the peak level just after administration and the accumulation of Ac-K at trough level. Although the sample was very small, this observation suggests there might be a mechanism of accumulation at trough that is not related to transient elevation of Ac-K levels. Because of the small sample size, we were not able to evaluate the relationship between Ac-K level and efficacy or safety in this study. Further studies are awaited to investigate the epigenetic mechanism contributing to therapeutic effects in BC patients.

### T-cell activation markers

It has been reported previously that ENT increases T-cell activation markers such as CD69 in PBMCs derived from lung cancer patients [34]. In Cohorts 1–3, weekly administration of ENT was associated with an increase in HLA-DR in CD4+ and CD8+ T-cells over time in the overall population. CD69 expression was also increased in some patients, particularly those with SD for ≥ 6 months. We plan to further verify whether increased expression of HLA-DR and CD69 is related to drug efficacy in future clinical trials.

### Efficacy

The mPFS of postmenopausal women with HR+/HER2− advanced or metastatic BC who received NSAIs as first-line therapy is approximately 9–10 months [35, 36]. The mPFS with second-line therapy tends to be shorter than with first-line therapy. For example, in the BOLERO-2 study, the mPFS in patients who received EXE monotherapy as second-line or later hormonal therapy was 4.1 months [4], and it was 4.21 months in 35 Japanese patients [37]. In ENCORE 301, the mPFS of EXE monotherapy in a similar setting was 2.3 months, which was shorter than in BOLERO-2, whereas that of EXE in combination with ENT was 4.3 months [20]. In this study, the mPFS of 12 patients receiving ENT + EXE as second-line or later hormonal therapy was 13.9 months. Generally, PFS cannot be compared directly between studies because it is affected by study setting and patient demographics. Nevertheless, it may be surprising that the mPFS observed in this study was longer than those in previous studies evaluating first-line hormonal therapy. One explanation is a good ECOG PS in patients: 0 in 91.7% of patients in this study, but only 63.0% in ENCORE 301 [20]. However, five of 12 patients had received ≥ 3 prior regimens of hormonal therapy in the advanced or metastatic setting, and these patients usually achieve a PFS of only a few months with hormonal therapies. The efficacy of 5 mg ENT weekly + EXE in Japanese patients who had previously received several endocrine therapies will be further clarified when the results of an ongoing, placebo-controlled, phase 2 study (NCT03291886) are available.

Because the results explicitly demonstrated that ENT had some positive effect on the efficacy of hormonal monotherapy, they support the current hypothesis that ENT can improve sensitivity to hormonal therapies in BC patients. To further elucidate the comprehensive biological effect underlying the efficacy of ENT, additional investigations and research are required into the complex network of cancer cells and the tumor microenvironment in terms of changes in the key signal transduction cascade and epigenetic modification.

Among six patients who achieved prolonged SD, four patients had received previous fulvestrant for advanced or metastatic BC. Although nonclinical and clinical evidence suggested the efficacy of ENT after the development of resistance to NSAIs, there were no published efficacy data after treatment with fulvestrant. These data are encouraging because ENT may be effective even after ER degradation therapy. Further implications may arise from the ongoing E2112 study. Given that no patients had previously received CDK4/6 inhibitors because these treatments were not approved in Japan at the time the trial started, we cannot evaluate their impact in this study. To further elucidate how to incorporate ENT into the current treatment paradigms of sequential hormonal therapies in combination with molecular targeted therapies, we await the results of the ongoing, placebo-controlled, phase 3 global and phase 2 Japanese studies.

Other HDACi with structures such as hydroxamic acid groups and cyclic peptides are being developed, mainly for blood cancers such as peripheral T-cell lymphoma, cutaneous T-cell lymphoma, and myeloma. Only tucidinostat, a class I selective HDACi with a similar benzamide structure to ENT, is being actively developed for use in BC. In the phase 3 ACE trial, tucidinostat + EXE demonstrated a significantly longer PFS compared with placebo + EXE, with AEs that were manageable despite occurring at a higher frequency [38]. Tucidinostat has already been approved in China, which suggests that class I selective HDACi may be useful for BC.

## Conclusions

This study demonstrated the tolerability of ENT 3 mg, 5 mg, and 10 mg as monotherapy followed by 5 mg once weekly in combination with EXE. AEs were consistent with those reported in previous studies and were manageable with appropriate measures, such as dose interruption and reduction, as well as prophylactic treatment. The clinical benefit of ENT in the second-line or later setting was observed, even after the use of fulvestrant. Furthermore, both an increase in Ac-K shortly after administration and the accumulation of Ac-K as a trough level were confirmed in this study.

Although limited by the small sample size, these results warrant further investigation to evaluate the current hypothesis that ENT can improve sensitivity to hormonal therapies.

## Supplementary Information

**Additional file 1.** Study design. Doses of ENT are shown in circles. *DLT* dose-limiting toxicity, *ENT* entinostat, *EXE* exemestane.

**Additional file 2. **Change in T-cell activation marker expression (MFI) on CD4+ and CD8+ T-cells in PBMCs of patients from Cohorts 1 to 3. Data are presented as the fold change relative to C0D1 on C0D2, C1D1, C1D8, and C2D1. No statistically significant changes were observed. **a** Change in HLA-DR expression on CD8+ T-cells. **b** Change in HLA-DR expression on CD4+ T-cells. **c** Change in CD69 expression on CD8+ T-cells. **d** Change in CD69 expression on CD4+ T-cells. **e‍** Change in ICOS expression on CD8+ T-cells. **f** Change in ICOS expression on CD4+ T-‍cells. *C* cycle number, *D* day number, *HLA-DR* human leukocyte antigen-DR, *ICOS* inducible T-cell costimulator, *MFI* median fluorescence intensity, *PBMC* peripheral blood mononuclear cell.

**Additional file 3. **Change in T-cell activation marker expression (MFI) on CD4+ and CD8+ T-cells in PBMCs of patients from Cohorts 1 to 3, stratified by PFS (< 6 months or ≥ 6‍ months). Data are presented as the fold change relative to C0D1 on C2D1. A statistically significant difference between PFS subgroups was observed for the change in CD69 expression on CD8+ T-cells (**c**), but not for other T-cell activation markers. **a** Change in HLA-DR expression on CD8+ T-cells. **b** Change in HLA-DR expression on CD4+ T-cells. **c‍** Change in CD69 expression on CD8+ T-cells. **d** Change in CD69 expression on CD4+ T-‍cells. **e** Change in ICOS expression on CD8+ T-cells. **f** Change in ICOS expression on CD4+ T-cells. *P = 0.037 (Wilcoxon test). *C* cycle number, *D* day number, *HLA-DR* human leukocyte antigen-DR, *ICOS* inducible T-cell costimulator, *MFI* median fluorescence intensity, *PBMC* peripheral blood mononuclear cell, *PFS* progression-free survival.

## Data Availability

The datasets generated and/or analyzed during the study sponsored by Kyowa Kirin will be available in the Vivli repository, https://vivli.org/ourmember/kyowa-kirin/ as long as conditions of data disclosure specified in the policy section of the Vivli website are satisfied.

## Abbreviations

Ac-K: Protein lysine acetylation
AE: Adverse event
AUC_0–168_: Area under the plasma concentration-time curve from time 0–168 h
BC: Breast cancer
CBR: Clinical benefit rate
CDK: Cyclin-dependent kinase
C_max_: Maximum plasma concentration
CR: Complete response
DLT: Dose-limiting toxicity
ECOG PS: Eastern Cooperative Oncology Group performance status
ENT: Entinostat
ER: Estrogen receptor
EXE: Exemestane
HDAC: Histone deacetylase
HDACi: Histone deacetylase inhibitor
HER2–: Human epidermal growth factor receptor 2-negative
HLA-DR: Human leukocyte antigen-DR
HR: Hazard ratio
HR+: Hormone receptor-positive
mPFS: Median progression-free survival
mTOR: Mammalian target of rapamycin
NSAI: Nonsteroidal aromatase inhibitor
OS: Overall survival
PBMC: Peripheral blood mononuclear cell
PD: Progressive disease
PFS: Progression-free survival
PK: Pharmacokinetics
PR: Partial response
RECIST: Response Evaluation Criteria in Solid Tumours
SAE: Serious adverse event
SD: Stable disease
