# Supplementary material for: Phase 1 trial of entinostat as monotherapy and combined with exemestane in Japanese patients with hormone receptor-positive advanced breast cancer
Source: BMC Cancer. 2021 Nov 24;21:1269. doi: 10.1186/s12885-021-08973-4 (PMC8611843; doi:10.1186/s12885-021-08973-4)

Additional file 1 (Additional file 1.pdf) Study design. Doses of ENT are shown in circles. *DLT* dose-limiting toxicity, *ENT* entinostat, *EXE* exemestane

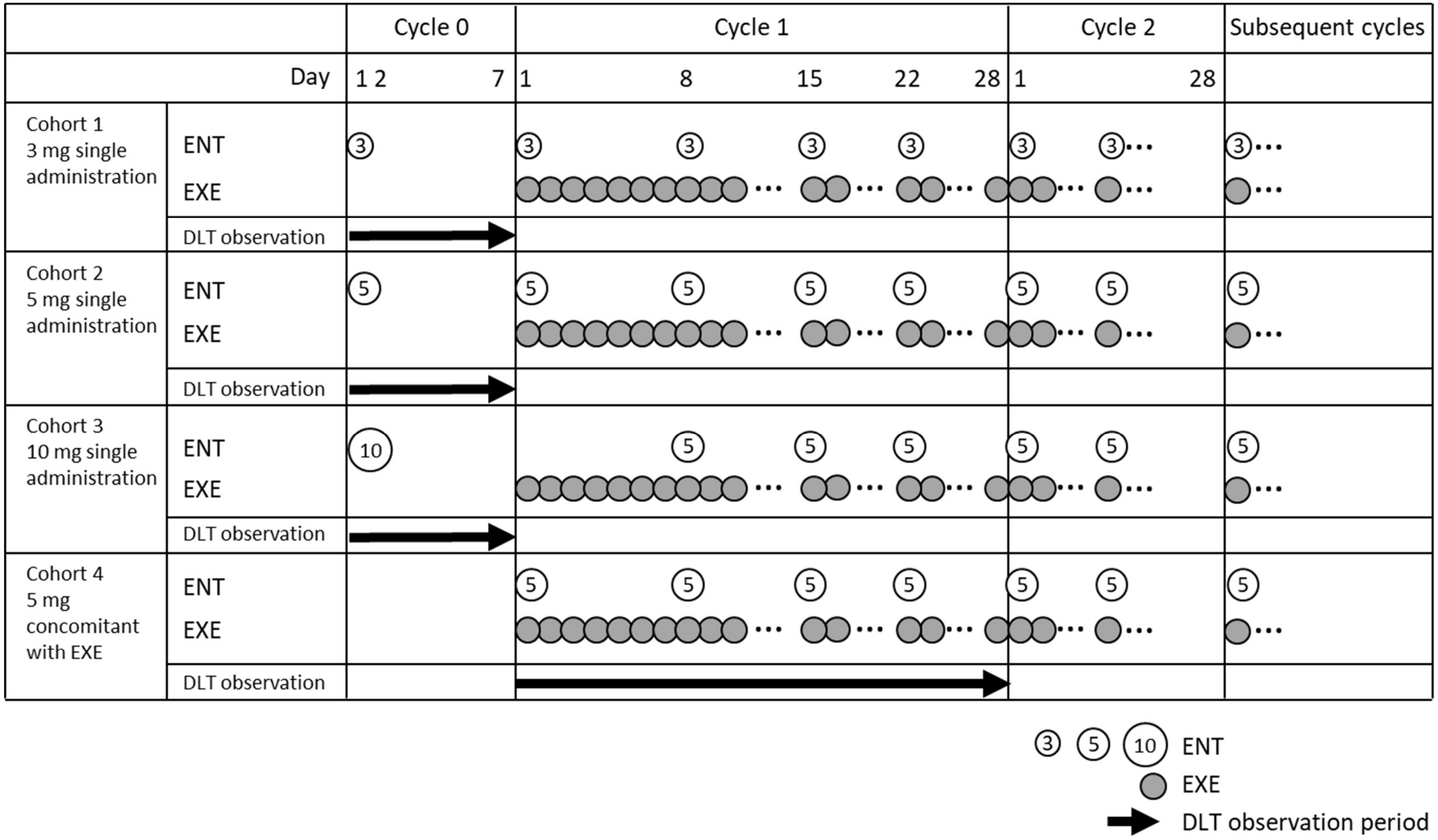

Supplement: Supplementary file 1 — Additional file 1. Study design. Doses of ENT are shown in circles. DLT dose-limiting toxicity, ENT entinostat, EXE exemestane. [file 12885_2021_8973_MOESM1_ESM.pdf]
