# Supplementary material for: Phase 1 trial of entinostat as monotherapy and combined with exemestane in Japanese patients with hormone receptor-positive advanced breast cancer
Source: BMC Cancer. 2021 Nov 24;21:1269. doi: 10.1186/s12885-021-08973-4 (PMC8611843; doi:10.1186/s12885-021-08973-4)
Supplement: Supplementary file 2 — Additional file 2. Change in T-cell activation marker expression (MFI) on CD4+ and CD8+ T-cells in PBMCs of patients from Cohorts 1 to 3. Data are presented as the fold change relative to C0D1 on C0D2, C1D1, C1D8, and C2D1. No statistically significant changes were observed. a Change in HLA-DR expression on CD8+ T-cells. b Change in HLA-DR expression on CD4+ T-cells. c Change in CD69 expression on CD8+ T-cells. d Change in CD69 expression on CD4+ T-cells. e‍ Change in ICOS expression on CD8+ T-cells. f Change in ICOS expression on CD4+ T-‍cells. C cycle number, D day number, HLA-DR human leukocyte antigen-DR, ICOS inducible T-cell costimulator, MFI median fluorescence intensity, PBMC peripheral blood mononuclear cell. [file 12885_2021_8973_MOESM2_ESM.pdf]

Additional file 2 (Additional file 2.pdf) Change in T-cell activation marker expression (MFI) on CD4+ and CD8+ T-cells in PBMCs of patients from Cohorts 1 to 3. Data are presented as the fold change relative to C0D1 on C0D2, C1D1, C1D8, and C2D1. No statistically significant changes were observed. **a** Change in HLA-DR expression on CD8+ T-cells. **b** Change in HLA-DR expression on CD4+ T-cells. **c** Change in CD69 expression on CD8+ T-cells. **d** Change in CD69 expression on CD4+ T-cells. **e** Change in ICOS expression on CD8+ T-cells. **f** Change in ICOS expression on CD4+ T-cells. *C* cycle number, *D* day number, *HLA-DR* human leukocyte antigen-DR, *ICOS* inducible T-cell costimulator, *MFI* median fluorescence intensity, *PBMC* peripheral blood mononuclear cell

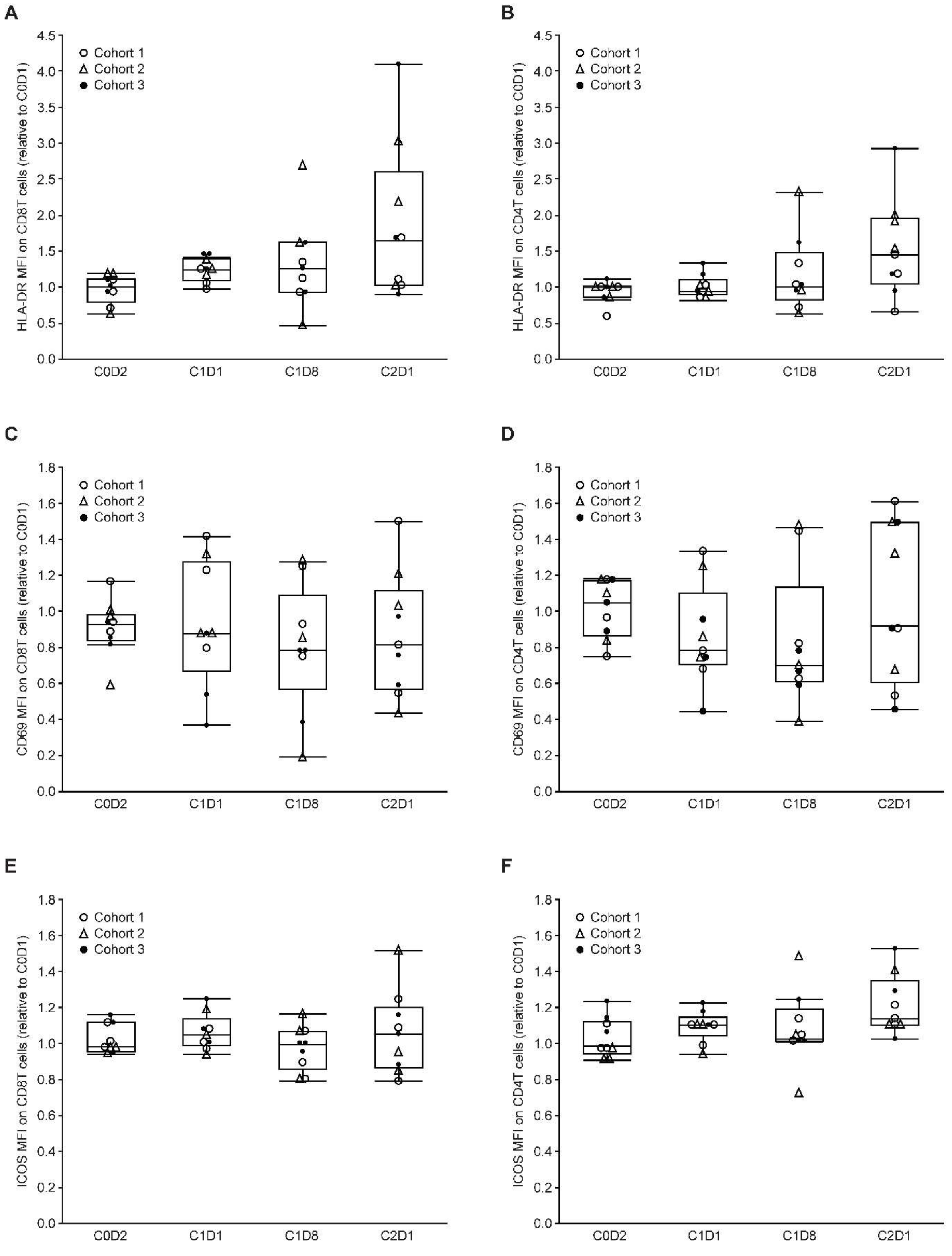
A
HLA-DR MFI on CD8T cells (relative to C0D1)
Cohort 1
Cohort 2
Cohort 3
0.0
0.5
1.0
1.5
2.0
2.5
3.0
3.5
4.0
4.5
C0D2
C1D1
C1D8
C2D1
B
HLA-DR MFI on CD4T cells (relative to C0D1)
Cohort 1
Cohort 2
Cohort 3
C0D2
C1D1
C1D8
C2D1
C
CD69 MFI on CD8T cells (relative to C0D1)
Cohort 1
Cohort 2
Cohort 3
0.0
0.2
0.4
0.6
0.8
1.0
1.2
1.4
1.6
1.8
C0D2
C1D1
C1D8
C2D1
D
CD69 MFI on CD4T cells (relative to C0D1)
Cohort 1
Cohort 2
Cohort 3
C0D2
C1D1
C1D8
C2D1
E
ICOS MFI on CD8T cells (relative to C0D1)
Cohort 1
Cohort 2
Cohort 3
C0D2
C1D1
C1D8
C2D1
F
ICOS MFI on CD4T cells (relative to C0D1)
Cohort 1
Cohort 2
Cohort 3
C0D2
C1D1
C1D8
C2D1
